# Investigation of the Relationship Between Neutrophil/Lymphocyte, Platelet/Lymphocyte, IgA/C3, IgA/C4, and C3/C4 Ratios and Proteinuria in Patients With Immunoglobulin A (IgA) Nephropathy

**DOI:** 10.7759/cureus.79060

**Published:** 2025-02-15

**Authors:** Yasemin Kirac, Murat Duranay

**Affiliations:** 1 Department of Nephrology, Faculty of Medicine, Kirikkale University, Kırıkkale, TUR; 2 Department of Nephrology, Ankara Training and Research Hospital, Ankara, TUR

**Keywords:** c3, c4, igan, neutrophil-to-lymphocyte ratio, platelet-to-lymphocyte ratio, proteinuria

## Abstract

Background

Immunoglobulin A nephropathy (IgAN) is the most common primary glomerular disease, characterized by IgA-containing immune complexes in the mesangium and mesangial cell proliferation. Proteinuria is a strong indicator of the progression of chronic kidney disease. This study aimed to evaluate the relationship between systemic inflammation markers, including the neutrophil-to-lymphocyte ratio (NLR), platelet-to-lymphocyte ratio (PLR), IgA/complement (C)3, IgA/C4, and C3/C4 ratios, and proteinuria in IgAN patients.

Methods

This retrospective study involved 38 patients diagnosed with IgAN between 2002 and 2011. We assessed various serum markers such as leukocyte count, NLR, PLR, C-reactive protein (CRP), C3, C4, IgA, and IgM, as well as proteinuria. The correlation between these markers and proteinuria was examined.

Results

The mean age of the patients was 37.8 ± 2 years, with 68.42% male. No significant correlation was found between the NLR and proteinuria (p=0.3) or CRP (p=0.3). However, a moderate positive correlation was observed between the NLR and sedimentation rate (R=0.38, p=0.07). Significant negative correlations were found between proteinuria and both urine pH (R=-0.5, p=0.002) and IgA/C4 ratio (R=-0.5, p=0.013). There were positive correlations between the PLR and C4 (R=0.45, p=0.02) and negative correlations between the PLR and IgA/C4 ratio (R=-0.56, p=0.003) and IgA/C4 and C3 (R=-0.48, p=0.008). No significant differences in proteinuria and leukocyte count were observed between the two sexes.

Conclusion

Although no significant relationship was found between the NLR, PLR, and proteinuria in IgAN, correlation between the IgA/C4 ratio and C4 warrants further investigations in larger studies.

## Introduction

Immunoglobulin A nephropathy (IgAN) is the most common primary glomerular disease worldwide [1]. The prevalence of IgAN is highest among individuals of East Asian descent and relatively rare in those of African descent [2]. This disparity may result from factors such as genetic susceptibility, differences in urine screening tests, access to nephrologists, biopsy indications, and the availability of immunofluorescence microscopy [3]. Approximately 20-40% of patients with IgAN may progress to end-stage renal disease (ESRD) within 20 years, posing a significant clinical and financial burden on global public health [4]. Autoimmunity and inflammation are key mechanisms in its pathophysiology. The process begins with the production of galactose-deficient immunoglobulin A (GD-IgA) in genetically predisposed individuals, which then interacts with autoantibodies [5]. The "multiple hit hypothesis" is used to explain IgAN's development: Hit 1: Overproduction of GD-IgA1 (IgA1 with galactose deficiency); Hit 2: Production of anti-glycan autoantibodies (IgG or IgA) recognizing GD-IgA1; Hit 3: Formation of circulating immune complexes; Hit 4: Accumulation of these immune complexes in the glomerular mesangium, activating the complement system, and initiating inflammation, fibrosis, and sclerosis. This leads to hematuria, proteinuria, glomerular and tubular sclerosis, and, ultimately, kidney dysfunction and failure [6,7]. 

Proteinuria is a symptom of kidney disease and its presence provides strong prognostic information [8]. According to Kidney Disease Improving Global Outcomes (KDIGO) 2021 guidelines, reducing proteinuria levels to below 1 g/day was recommended as a reasonable treatment goal for IgAN patients [9]. However, the KDIGO 2024 Clinical Practice Draft Guidelines for the treatment of IgAN and IgA vasculitis were recently made available for public rewove. In this updated guideline, the treatment goal for IgAN patients is to reduce proteinuria to <0.5 g/day (preferably <0.3 g/day) [10]. Approximately 50% of patients with proteinuria >1 g/day and a decline in kidney function will progress to ESRD within 10 years [11]. A study conducted by Chinese researchers found that IgAN patients with daily proteinuria between 0.5 and 1 g had a 10-year dialysis-free survival rate of 95% and a 20-year dialysis-free survival rate of 89%. It has also been found that a reduction in proteinuria is associated with a significant decrease in the risk of kidney dysfunction [12].

As new inflammatory factors, the neutrophil-to-lymphocyte ratio (NLR) and platelet-to-lymphocyte ratio (PLR) are inflammatory biomarkers with prognostic value in various disease processes [13,14]. The NLR reflects the dynamic relationship between innate (neutrophils) and acquired (lymphocytes) immune responses in many pathological conditions. It is influenced by factors such as age, diet, medication use, coronary heart disease, stroke, diabetes, obesity, psychiatric conditions, solid organ cancers, anemia, and stress. The normal range for the NLR is between 1 and 2, with values above 3 or below 0.7 considered pathological. An NLR range of 2.3-3 may indicate early pathological conditions such as cancer, atherosclerosis, infection, inflammation, psychiatric disorders, and stress [15]. Recent studies have shown associations between the NLR and PLR with the phenotype and prognosis of kidney diseases, including chronic kidney disease (CKD), acute kidney injury, and rapidly progressive glomerulonephritis [16,17].

In IgAN, the complement system is activated through the alternative and lectin pathways. A study on a large patient cohort found that a decrease in serum complement 3 (C3) levels is an independent risk factor for a doubling of creatinine and for ESRD [18].

Factors such as heavy proteinuria, reduced kidney function, hypertension, and glomerular sclerosis are associated with poor outcomes. However, there are no serological tests to assess disease activity. While IgA accumulation is a key diagnostic feature, C3 accumulation is also frequently observed [19]. Some studies suggest that the serum IgA/C3 ratio may predict the progression of IgAN (more than a 50% reduction in the estimated glomerular filtration rate (eGFR)) more accurately than serum C3 or IgA levels alone [20]. Other studies propose that serum complement 4 (C4) could serve as a marker for IgAN progression [21,22]. This study aimed to investigate the relationship between inflammatory markers NLR, PLR, IgA/C3, IgA/C4, and C3/C4 ratios and proteinuria in patients with IgAN.

## Materials and methods

This retrospective study was conducted following ethical approval (decision number: 246/2024, dated October 24, 2024) between January 1, 2002, and February 1, 2011, involving 38 patients who were diagnosed with IgA nephropathy through kidney biopsy at the Nephrology Clinic of Ankara Training and Research Hospital. This study included male and female patients over the age of 18 who were diagnosed with IgA nephropathy through kidney biopsy. Patients with a history of diabetes mellitus, malignancy, chronic infectious diseases, chronic liver or gastrointestinal diseases, or autoimmune disorders were excluded from the study. Before kidney biopsy, all patients used the RAS blocking medication for at least three months. None of the patients used immunosuppressive drugs or SGLT2 inhibitors.

Patient records were reviewed for age, gender, and serum leukocyte count, neutrophil count, platelet count, lymphocyte count, CRP, sedimentation rate, C3, C4, IgG, IgA, and IgM levels, urine pH, urine density, spot urine proteinuria, and 24-hour urine protein levels. NLR, PLR, IgA/C3, IgA/C4, and C3/C4 ratios were calculated. 

Recording the patient's age, gender, complete blood count, serum complement C3, C4, IgG, IgA. and IgM levels, complete urinalysis, spot urine proteinuria, and 24-hour urine protein levels simultaneously with kidney biopsy and NLR was planned to calculate PLR, IgA/C3, IgA/C4, and C3/C4 ratios.

Statistical analysis

Statistical analysis was performed using IBM SPSS Statistics for Windows, Version 27 (Released 2020; IBM Corp., Armonk, New York, United States). The normality of the data distribution was assessed using the Shapiro-Wilk test. Continuous variables were compared between two groups using either the Student’s t-test or the Mann-Whitney U test, depending on the distribution. Data were presented as mean ± SEM. Correlation analysis between proteinuria and NLR, PLR, IgA/C3, IgA/C4, and C3/C4 ratios was conducted using the Spearman correlation test. A p-value of <0.05 was considered statistically significant. 

## Results

The mean age of the 38 patients included in our study was 37.8 ± 2 years. Twelve of the cases were female (31.57%), and 26 were male (68.42%). The laboratory parameters of the patients are presented in Table 1.

**Table 1:** Demographic and Laboratory Data of Patients with IgA Nephropathy The normality of the data distribution was assessed using the Shapiro-Wilk test Continuous variables were compared between two groups using either Student’s t-test or the Mann-Whitney U test, depending on the distribution Data were presented as mean ± SEM CRP, C reactive protein; NLR, Neutrophil-to-lymphocyte ratio; PLR, Platelet-to-lymphocyte ratio; IgA, Immunoglobulin A; IgG, Immunoglobulin G; IgM, Immunoglobulin M; C3, complement 3; C4, complement 4

Parameter	Mean± SEM
Age (year)	37.8±2
Gender (female/male)	12/26
Urine pH (no unit)	5.8±0.15
Urine density (no unit)	1017±1.1
Proteinuria (mg/day)	1887±309
Sedimentation (mm/h)	22.1±3.1
CRP (mg/dL)	1.1±0.5
Hemoglobin (g/dL)	14.4±0.3
Hematocrit (%)	41±0.9
Leukocyte count x1000	8105±424
Neutrophil countx1000	5233±373
Platelet count x1000	254±69
Lymphocyte x 1000	2054±127
NLR	2.8 ±0.3
PLR	125.7±10.7
IgG (mg/dL)	1242±74
IgA (mg/dL)	319±20
IgM (mg/dL)	150±26
C3 (mg/dL)	124±3
C4 (mg/dL)	25±1
C3/C4	5.3±0.3
IgA/C3	2.6±0.2
IgA/C4	13.7±0.9

In the correlation analysis conducted, a strong negative correlation between proteinuria and pH was found (R=0.5, p=0.002), while no correlation was observed between proteinuria and NLR or PLR. We observed a positive correlation between C3 and C4 levels (R=0.48, p=0.007).

While no correlation was observed between proteinuria and the IgA/C3 ratio, a strong negative correlation was found between proteinuria and the IgA/C4 ratio (R=-0.5, p=0.013) (Table 2).

**Table 2:** Correlation Analysis Correlation analysis between proteinuria and NLR, PLR, IgA/C3, IgA/C4, and C3/C4 ratios was conducted using the Spearman correlation test A p-value of <0.05 was considered statistically significant NLR, Neutrophil-to-lymphocyte ratio; PLR, Platelet-to-lymphocyte ratio; IgA, Immunoglobulin A; C3, complement 3; C4, complement 4

Parameter	Parameter	Correlation Coefficient	P value
Proteinuria (mg/day)	Urine pH	-0.5	0.002
Proteinuria (mg/day)	NLR	0.04	0.8
Proteinuria (mg/day)	PLR	-0.05	0.7
Proteinuria (mg/day)	IgA/C3	-0.01	0.7
Proteinuria (mg/day)	IgA/C4	-0.5	0.013
IgA/C3	IgA/C4	0.7	<0.001
IgA/C3	C3/C4	-0.4	0.046
PLR	C4	0.5	0.02
PLR	IgA/C4	-0.56	0.003
IgA/C4	C3	-0.48	0.008

When we divided our patients into two groups based on the IgA/C3 ratio (<3.3 and >3.3) [20], the group with the higher ratio had a higher age and a lower estimated glomerular filtration rate (eGFR), with no significant difference in gender distribution (Table 3).

**Table 3:** Subgroup Analysis Parameter comparison according to NLR, IgA/C3, IgA/C4, and C3/C4 binary grouping. Either the Mann-Whitney U test or independent t-test was used according to data distribution normality. A p-value of <0.05 was considered statistically significant. NLR, Neutrophil-to-lymphocyte ratio; GFR, Glomerular filtration rate; IgA,Immunoglobulin A; IgM, Immunoglobulin M; C3, complement 3; C4, complement 4

	NLR<2.4	NLR≥2.4	p	IgA/C3<3.3	IgA/C3≥3.3	p	C3/C4<5.1	C3/C4≥5.1	p
Age(year)	40.2± 3.2	40.3± 3.1	0.9	38.1± 2.4	43.6± 4.5	0.3	39.7± 2.2	37.1± 4.3	0.6
Proteinuria (mg/day)	1318± 205	2422± 693	0.1	1951,4± 37.9	1744.8± 910.8	0.8	2178± 500	1422± 406.4	0.3
GFR (ml/min)	62.3± 5.9	51.5± 6.5	0.2	63.5± 5	45± 6.5	0.1	53.4± 4.7	72.7± 7.5	0.03
C3 (mg/dL)	119.4± 4.7	130.4± 5.6	0.1	127.4± 4.2	110.9± 5.9	0.08	123.1± 4.7	127.1± 6.5	0.6
C4 (mg/dL)	21.5± 1.5	28.7± 2.2	0.01	23.5± 1.5	29.6± 3.8	0.08	28.3± 1.6	19.7± 1.6	0.001
IgM (mg/dL)	146.1± 26.4	175± 57.3	0.6	150.9± 31.4	151.5± 55.9	0.9	166± 44.5	125.3± 17.5	0.5
IgA/C3	2.8± 0.3	2.5± 0.3	0.6	-	-	-	2.9± 0.3	2.2± 0.2	0.03
IgA/C4	15.8± 1.3	12± 1.3	0.053	12.8± 0.9	17.2± 1.9	0.053	13.1± 1.2	14.6± 1.4	0.4

A strong positive correlation was found between the IgA/C3 ratio and the IgA/C4 ratio (R=0.7, p<0.001), and a strong negative correlation was found between the IgA/C3 ratio and the C3/C4 ratio (R=-0.4, p=0.046) (Table 2). We observed a positive correlation between PLR and C4 (R=0.45, p=0.02), while a strong negative correlation was found between PLR and IgA/C4 (R=-0.56, p=0.003). A strong negative correlation was also observed between IgA/C4 and C3 (R=-0.48, p=0.008) (Table 2).

When we divided our study population into two groups based on NLR (<2.4 and ≥2.4) [16], no significant differences were observed between the two groups for GFR, IgA/C3, C3, and IgM parameters. However, significant differences were noted for IgA/C4 (p=0.053) and C4 (p=0.01) parameters. Additionally, in the group with NLR ≥2.4, GFR was lower, and although the levels of C3, C4, and IgM were not statistically significant, they were higher (Table 3).

When we divided our study population into two groups based on the C3/C4 ratio (<5.1 and ≥5.1) [22], the group with the lower ratio had a lower GFR (53±4.7 vs 72±7 ml/min, p=0.03) and a higher proteinuria level (2178±500 vs 1422±406 mg/day, p=0.2) (Table 3).

## Discussion

In our retrospective study, we found no correlation between proteinuria and the NLR or PLR. However, we identified a positive correlation between the PLR and C4, as well as a strong negative correlation between the PLR and IgA/C4. Additionally, we observed a strong negative correlation between IgA/C4 and C3, a strong positive correlation between IgA/C3 and IgA/C4, and a strong negative correlation between the IgA/C3 ratio and the C3/C4 ratio. Considering the role of inflammation and complement in the pathogenesis of IgAN, we believe that our data hold significant value in identifying guiding markers for the monitoring and management of proteinuria, which is a prognostic indicator of the disease.

The NLR is a simple calculation that easily assesses the inflammatory status. In a study conducted by Forget et al., in healthy adults, the mean NLR value was 1.65 [23]. In a study by Azab et al. in the U.S. with 9,427 participants, the mean NLR was 2.15. The mean NLR values for non-Hispanic Black and Hispanic participants were 1.76 and 2.08, respectively, while for non-Hispanic White participants, the value was 2.24, which was significantly higher when compared to these groups (p<0.0001). The study emphasized that these racial differences in the NLR are associated with chronic diseases and that different cut-off points should be established based on race. Additionally, it highlighted that there are racial differences in the inflammatory response to environmental and behavioral risk factors [24].

Previous studies have shown that the NLR is an independent marker for poor kidney prognosis in IgAN and adult IgA vasculitis. In a study by Li et al., patients with IgAN were divided into two groups using an NLR cut-off value of 2.4. Patients in the high NLR group exhibited a lower GFR, higher proteinuria, a higher incidence of hypertension, and more severe pathological lesions [16]. In our study, although there was no statistically significant difference, patients with NLR ≥2.4 had lower GFR and higher levels of C3, C4, and IgM.

In a study by Wang et al., the NLR was proposed as an independent prognostic factor for progression to ESRD in patients with IgAN. In a subgroup analysis examining the relationship between NLR and stages of CKD and 24-hour urinary protein in patients with IgAN, it was shown that a high NLR was significantly associated with the renal prognosis of patients with IgAN, particularly predictive in those with advanced-stage CKD or 24-hour urinary protein >1 g/day [25]. Due to the retrospective nature of our study, we were unable to make prognostic interpretations.

In the study by Chang et al., it was observed that among female patients with IgAN, a PLR >137 was associated with poor prognosis compared to PLR ≤137 after adjustments for age, hemoglobin, serum albumin, creatinine, eGFR, proteinuria, and immunosuppressive treatments. It was reported that patients in the PLR ≤137 group with a baseline eGFR <60 mL/min/1.73 m² had better kidney survival. Interestingly, in male patients, no differences in renal outcomes were observed according to PLR grouping in those with a baseline eGFR >60 mL/min/1.73 m² and those with baseline proteinuria ≤ 1g/day [17]. In our study, the average PLR was 125.7. While there was no correlation between the PLR and proteinuria, we found a positive correlation between PLR and C4. This discrepancy in results may arise from the small sample size of our population.

According to current guidelines, there is no accepted cut-off value for serum IgA levels in the diagnosis of IgAN, but elevated serum IgA is noteworthy. In a study by Tomino et al., it was stated that when using the CRM470 method, a nephelometric immunoassay accepted by the IFCC for serum IgA levels, an IgA level above 315 mg/dl could be considered a diagnostic criterion [26]. In our population, the mean IgA level was 319 mg/dl. Geographic and ethnic differences in studies should be kept in mind when interpreting these values.

In a study by Tomino et al. involving 306 patients with glomerulonephritis and 418 healthy adults, the potential of the IgA/C3 ratio for diagnosing IgAN was investigated. Out of these 306 patients, 195 had IgAN, while 111 had nephropathy other than IgAN. The study found that a cut-off value of 2.14 for the IgA/C3 ratio had a sensitivity of 79%, a specificity of 61%, and an overall accuracy of 73% for predicting IgAN diagnosis prior to kidney biopsy [27]. In our study, the average IgA/C3 ratio was 2.6. Due to the small size of our population and the lack of treatment and follow-up data, we cannot contribute any new findings beyond those presented in the study by Tomino et al.

Maeda et al. suggested that serum IgA and C3 levels measured before kidney biopsy could predict the clinical course of IgAN, proposing that serum IgA levels above 315 mg/dl or serum IgA/C3 ratios above 3.01 could serve as standards for diagnosis. It has been reported that the serum IgA/C3 ratio can be used to predict the prognosis of IgAN [28]. In our population, the average IgA/C3 ratio was 2.6, and the serum IgA level was 319 mg/dl. Our IgA/C3 ratio falls below the cut-off values suggested by Maeda et al., while our mean IgA level is above the cut-off, which may be due to ethnic differences. Larger population studies will help clarify this situation.

In a multicenter, retrospective cohort study by Torikoshi et al., the predictive power of the serum IgA/C3 ratio for progression was investigated in 718 patients diagnosed with IgAN. They reported that an IgA/C3 ratio ≥3.3 predicted a doubling of serum creatinine levels with a sensitivity of 61.4% and a specificity of 55.2%. When IgAN cases were divided into two groups based on the IgA/C3 ratio (≥3.3 and <3.3), no difference was observed in terms of proteinuria; however, the high ratio group showed higher age, serum creatinine levels, and a greater proportion of males. Among these 718 patients, in the 63 whose values were monitored throughout the study, those with a decrease in IgA/C3 ratio greater than 15% had a higher rate of disease remission [20]. In our study, the average IgA/C3 ratio was 2.6. In our small population, no correlation was observed between proteinuria and the IgA/C3 ratio; however, when we divided patients into two groups based on IgA/C3 ≥3.3 and <3.3, there were no significant differences between the groups in terms of gender and proteinuria, while the high ratio group had a higher age and lower eGFR.

In another study by Tringali et al., it was shown that patients with IgAN in the group with low C3 and C4 levels had higher proteinuria levels and more severe kidney damage at the time of diagnosis. The same study also observed a positive correlation between C3 and C4 levels, emphasizing that IgAN could be related to systemic complement consumption. Additionally, C3 and C4 levels showed a negative correlation with serum IgM levels, suggesting that the role of IgM needs further investigation. When patients were divided into three groups based on serum C3 levels (<90 mg/dl, 90-140 mg/dl, and >140 mg/dl), the low group had lower eGFR levels, while the medium group exhibited lower proteinuria. No relationship was observed between C3 levels and IgA or IgG levels, but both C3 and C4 were found to be associated with eGFR in a multiple linear regression model [29]. In our study, due to the small population size, we did not categorize patients based on C3 levels. Our mean C3 level was 124 mg/dl, which corresponds to the mean accepted value. Similar to the findings of Tringali et al., we observed a positive correlation between C3 and C4 levels in our study. However, we did not find a correlation between IgM levels and C3 or C4 levels in our population.

In a study by Zhu et al. conducted in 2014, it was reported that low C4 levels at the time of diagnosis, but with less severe lesions at biopsy, were associated with worse outcomes during follow-up, rendering biopsy interpretation insufficient for prognosis prediction. They explained the contradiction between low C4 levels, mild kidney damage, and poor prognosis as follows: C4 binds to IgM to clear immune complexes. In cases of C4 deficiency, these complexes accumulate in the kidneys. Low C4 levels are associated with increased kidney IgM accumulation, which shows a negative relationship between serum C4 and kidney IgM accumulation. Additionally, the IgM accumulating in the kidneys can lead to severe kidney damage, such as heavy proteinuria, crescent formation, and increased mesangial matrix. C4 deficiency may lead to insufficient activation of the lectin pathway, which could help protect glomeruli from initial damage during kidney biopsy. However, accumulated IgM and immune complexes that cannot be cleared by C4 may cause chronic glomerular damage over time [21]. In our study, we did not observe a correlation between C4 and IgM levels. This difference in results may stem from ethnic variations and the small sample size of our population.

While there is no study showing the relationship between the IgA/C4 ratio and proteinuria in patients with IgAN, our study found a mean IgA/C4 ratio of 13.7 and a strong negative correlation between proteinuria and the IgA/C4 ratio. Our findings require further support from additional studies.

In another study by Pan et al., it was noted that the C3/C4 ratio at the time of diagnosis predicted kidney prognosis more effectively than serum C3 or C4 levels alone. Patients with a low C3/C4 ratio had significantly lower 5- and 10-year kidney survival rates, and these patients may benefit from aggressive immunosuppressive therapies [30]. Since our study utilized cross-sectional data, we do not have prognostic information. New studies that track both treatment data and prognosis will help clarify this topic.

In a study by Zhang et al., patients were divided into four groups based on the C3/C4 ratio: C3/C4 ratio <3.6, 3.6 ≤ C3/C4 ratio <4.2, 4.2 ≤ C3/C4 ratio <5.1, and C3/C4 ratio ≥5.1. Statistical differences in the GFR and proteinuria were observed among the four groups. It was found that the lower the C3/C4 ratio, the higher the proteinuria and the more severe the chronic kidney damage [22]. In our study, the mean C3/C4 ratio was 5.3. When we intended to categorize patients into two groups based on whether their C3/C4 ratio was below or above 3.6, we did not group them since there was only one case with a ratio <3.6. When we divided our population into two groups based on our mean C3/C4 ratio value of <5.1 and ≥5.1, the group with <5.1 had a significantly lower eGFR. Although there was no statistically significant difference between the two groups, the low-ratio group had higher proteinuria.

This study has the following limitations: it is a retrospective study, has a small population size, and lacks prognostic data.

## Conclusions

In this retrospective study, no significant correlation was found between proteinuria and the NLR or PLR. However, we identified a positive correlation between the PLR and C4, and a negative correlation between the PLR and IgA/C4. Additionally, a negative correlation was observed between IgA/C4 and C3, and a positive correlation between IgA/C3 and IgA/C4. A strong negative correlation was also found between the IgA/C3 ratio and the C3/C4 ratio. These findings support the role of inflammation and the complement system in the pathogenesis of IgAN and suggest the potential for novel biomarkers to aid in the monitoring of proteinuria.
